# Evaluating the Knowledge, Attitudes, and Practices of Saudi Arabian Parents Regarding Red Flags in Developmental Milestones

**DOI:** 10.7759/cureus.52769

**Published:** 2024-01-23

**Authors:** Samer A Alzahrani, Abdullah M Alzahrani, Abdullah A Alsalem, Norah K Almudaymigh, Mohammed A Alghamdi, Roaa S Alzahrani, Omar A Aldaham, Deemah S AlHuraish, Rahaf T Alharbi, Rema F Alharbi, Mohammed M Alzahrani

**Affiliations:** 1 Pediatrics, Al-Baha University, Al-Baha, SAU; 2 Clinical Pharmacy, Al-Noor Specialist Hospital, Mecca, SAU; 3 College of Medicine, King Saud University, Riyadh, SAU; 4 College of Medicine, Alfaisal University, Riyadh, SAU; 5 Pediatrics, Al Aziziya Hospital, Jeddah, SAU; 6 General Practice, Al-Baha Health Affairs, Al-Baha, SAU; 7 Respiratory Therapy, King Saud bin Abdulaziz University for Health Sciences, Alahsa, SAU; 8 College of Medicine, Imam Abdulrahman Bin Faisal University, Dhahran, SAU; 9 Medicine, University of Tabuk, Tabuk, SAU; 10 Medicine, Al-Rayan College of Medicine, Al-Madinah al-Munawwarah, SAU; 11 Medicine, Al-Baha Faculty of Medicine, Al-Baha University, Al-Baha, SAU

**Keywords:** practices, red flags, attitudes, knowledge, developmental milestones, saudi arabian parents

## Abstract

Background

Parents serve a fundamental role in monitoring developmental milestones and identifying potential delays during early childhood, enabling timely interventions. However, previous studies in Saudi Arabia have shown limited awareness among parents regarding age-specific norms and red flags across developmental domains. This knowledge gap can severely impact the detection and management of abnormalities. Hence, a comprehensive understanding of Saudi parental knowledge, attitudes, and practices concerning childhood developmental trajectories is imperative.

Methodology

This cross-sectional study assessed developmental milestone awareness, beliefs, and behaviors among Saudi parents. A sample of 1,052 parents completed a validated 38-item questionnaire covering knowledge, attitudes, practices, and demographics. Knowledge was assessed across developmental domains using 22 multiple-choice questions, with scores categorized as excellent (≥75%), good (50%-75%), fair (40%-50%), or poor (≤39%). Attitudes and practices were captured on five-point Likert scales. Descriptive and chi-square analyses were conducted using IBM SPSS Statistics for Windows, Version 28.0 (IBM Corp., Armonk, NY).

Results

The majority of participants were females (844, 80.2%), with a mean age of 38.8 years. Serious knowledge inadequacies existed regarding developmental timelines across domains, especially motor milestones like crawling (93, 29.4% correct), sitting unsupported (45, 27.6%), pointing at objects (278, 26.4%), and responding to names (440, 41.8%). Overall, 2 (0.2%) participants showed excellent and 281 (26.7%) showed good understanding, while 490 (46.6%) had poor knowledge. Significant sociodemographic variations were observed, with women and experienced parents demonstrating greater awareness (*P *< 0.001). Despite knowledge gaps, 542 (51.5%) actively sought developmental information themselves, and over 50% trusted pediatric guidance. Most participants expressed a willingness to undergo screenings if risk factors existed and reported spending a considerable amount of daily interaction time with their children, focusing on developmental needs.

Conclusions

Critical developmental milestone knowledge shortfalls and selective attitudes persist among Saudi parents, warranting public education and physician-parent communication that enhance interventions to enable impactful developmental monitoring and prompt responses to abnormalities in a timely manner.

## Introduction

The child's mental and physical development is greatly influenced by their early years, and parents and physicians are the two main pillars that ensure that youngsters grow up healthy [1]. Children are initially assessed for developmental delays in the four fundamental domains of language and social, gross motor, and fine motor skills. Following the discovery of the delays, a more thorough assessment is conducted in the areas of cognition, language, motor abilities, and social and emotional behavior. A child's advancement in every aspect of human functioning is referred to as "development" in the definition [2]. Parents need to be the most knowledgeable about children's needs and their developmental milestones in all areas, including language, motor skills, and social and behavioral development, as they are typically the primary caregivers and spend the most time with children. Parents' worries about their child's growth might be a good indicator of true developmental delays [3].

Red flags in developmental milestones can be discovered through three different channels: parents, doctors during routine check-ups, or teachers who detect something [4]. Pediatric care often includes assessing young children's mastery of particular developmental milestones [5,6]. Guidelines with lists of specific milestones and the ages at which most children should pass are published by organizations like the Centers for Disease Control and Prevention (CDC) and Bright Futures, a health promotion program sponsored by the American Academy of Pediatrics. In addition, the CDC advises parents to "act early by talking to their physician" if certain milestones have not been met [7].

Parents must understand the necessity to take their child to the doctor for routine check-ups and right away if they see a warning sign. Previous literature has highlighted limited parental knowledge about children's developmental milestones in Saudi Arabia. In a cross-sectional study conducted in 2023 on parental knowledge of children's motor development in Saudi Arabia, it was found that less than 50% of participants correctly answered the developmental milestone questions, indicating limited parental knowledge [8]. Similarly, another study conducted in the Asser region by Habbash et al. investigated parental knowledge of children's developmental milestones. The study revealed that only 29 (7.7%) participants had outstanding knowledge, while 141 (37.6%) participants had poor knowledge. Additionally, 180 (48%) participants had a sufficient degree of knowledge [9]. Furthermore, a cross-sectional study conducted in the Eastern Province of Saudi Arabia from January to February 2020 revealed that only 51.8% of participants were aware of developmental milestones. The study found statistically significant associations between mothers' education and parenting (*P* = 0.015) and their age (*P* = 0.001). Parenting and planned pregnancy also showed significant differences (*P* = 0.044) [10]. These studies collectively indicate a lack of adequate parental knowledge about children's developmental milestones in Saudi Arabia. Therefore, in this study, we aimed to assess parents' knowledge, attitudes, and practices regarding red flags of developmental milestones in Saudi Arabian children. Also, to identify any sociodemographic factors that may influence parents' knowledge related to developmental milestones in Saudi Arabian children.

## Materials and methods

Study design

This study employed a cross-sectional design to assess the level of knowledge, attitude, and practices of parents regarding the red flags of developmental milestones in children in Saudi Arabia.

Study setting: participants, recruitment, and sampling procedure

Our target population in this study included parents residing in Saudi Arabia who had at least one child or were planning to have one. This population was of interest as parents played a crucial role in monitoring their children's development and detecting potential delays or red flags in developmental milestones. The study was conducted in Saudi Arabia from November 2023 to December 2023, using a random sample technique. The study took place in various settings within Saudi Arabia that were accessible to parents, including community centers and schools. Additionally, different social media apps such as WhatsApp, Telegram, and X App were utilized. These settings were critical as they provided opportunities to recruit a diverse sample of parents. Participants were selected from different regions of Saudi Arabia to ensure a representative sample and to account for potential regional variations in knowledge, attitudes, and practices.

Inclusion and exclusion criteria

This study included parents of children or those planning to have children as the target population. The inclusion criteria encompassed parents who were citizens of Saudi Arabia. The exclusion criteria included individuals who were not citizens of Saudi Arabia, parents working as healthcare providers, and participants unwilling to take part in the study.

Sample size

Out of the initial pool of 1,210 participants, a total of 1,052 individuals met the criteria and were included in the study. However, 158 participants were excluded from the study. Among the excluded participants, 156 individuals worked as healthcare providers and two were unwilling to participate. The minimum required sample size for this study, as calculated using the Raosoft website, was determined to be 377 participants. This calculation was based on a 95% confidence level, a margin of error set at 5%, and an assumed distribution of responses at 50%.

Data collection and instrument (data collection technique and tools)

The tool used by Kumar et al. [11] was adopted with permission for this study. The questionnaire comprised four sections, namely, demographics, knowledge, parental practices, and parental attitude regarding developmental milestones. A total of 38 questions were included in the questionnaire. To facilitate data collection, we utilized Google Forms, an online survey platform, ensuring easy accessibility and efficient management of responses. Participants were provided with a link to the Google Forms questionnaire, enabling them to complete the survey electronically. The demographic section of the survey asked questions about gender, age, place of residence, number of children, level of education, and occupation. The knowledge assessment was divided into four parts, with each part containing questions on language, social, and gross and fine motor milestones. Each multiple-choice question in the knowledge section asked participants about developmental milestones, with 22 questions covering all domains and four options provided for them to choose from. Attitudes about red flags of developmental milestones were assessed using five questions, with response options including *strongly agree*, *agree*, *neutral*, *disagree*, and *strongly disagree*. Parental practices also consisted of five questions, with answer choices being either multiple-choice format or agree/disagree options. The scores for knowledge were aggregated to yield a total score based on the 22-item assessment. Parental knowledge scores were further categorized as *excellent* (≥75%), *good* (50%-75%), *fair* (40%-50%), and *poor* (≤39%). The scores were considered *acceptable* if they were ≥50% for each knowledge domain.

Analyses and entry method

The administration of data was performed using Microsoft Excel 2021. A structured Excel spreadsheet was created to enable the systematic entry of acquired information. Careful accuracy was maintained during the data entry process to ensure data integrity. After entering the data into Excel, a thorough data cleaning was conducted to verify the accuracy and quality of the data. The acquired data were coded before being subjected to statistical analysis using IBM SPSS Statistics for Windows, Version 28.0 (IBM Corp., Armonk, NY). During the analysis phase, participants' demographic information, along with their levels of knowledge, attitude, and practices regarding red flags of developmental milestones, was summarized using descriptive statistics. The participants' knowledge was evaluated using frequencies and percentages. Chi-square tests were also used to investigate any associations between variables and assess the extent of knowledge and awareness of developmental milestones. The level of significance was set at 0.05 to ensure the identification of statistically significant associations.

Ethical approval

Approval for the study was secured from the Scientific Research and Ethics Committee, Faculty of Medicine, Al-Baha University, with the designation REC/PEA/BU-FM/2023/101. To ensure informed consent from every participant and maintain the confidentiality of acquired data, the questionnaire featured an introductory question. Rigorous labeling and handling procedures were implemented to safeguard the information's security, and no personally identifiable information pertaining to participants was collected.

## Results

Sociodemographic data

Table 1 summarizes the sociodemographic data of the total participants (*n *= 1,052). The average age of the participants was 38.8 years, with a standard deviation of 11.6 years. The majority of participants were female (844, 80.2%), and most had a bachelor's degree or diploma (753, 71.6%). The participants were distributed across different regions, with the Eastern Region having the highest proportion (264, 25.1%). In terms of the number of children, the largest group consisted of participants with four or more children (477, 45.3%). The analysis of sociodemographic data among parents in the study revealed significant associations between gender (*P* < 0.001), whereas females exhibit a higher level of knowledge compared to males. The number of children was found to be significantly associated with parents' knowledge of developmental milestones' red flags (*P* = 0.001), whereas parents who had children tended to have better knowledge of developmental milestones' red flags.

**Table 1:** Summary of sociodemographic characteristics and associations among participants (n = 1,052).

Sociodemographic data				Knowledge status				*P*-value
				Unacceptable		Acceptable		
		Count, *n*	%	Count, *n*	%	Count, *n*	%	
Age	<24 years	140	13.3	109	77.9	31	22.1	0.581
	24-34 years	239	22.7	173	72.4	66	27.6	
	35-44 years	316	30.0	223	70.6	93	29.4	
	45-54 years	247	23.5	182	73.7	65	26.3	
	More than 55 years	110	10.5	82	74.5	28	25.5	
Gender	Male	208	19.8	185	88.9	23	11.1	<0.001
	Female	844	80.2	584	69.2	260	30.8	
Educational status	Primary	12	1.1	10	83.3	2	16.7	0.696
	Intermediate	29	2.8	19	65.5	10	34.5	
	High school	163	15.5	118	72.4	45	27.6	
	Bachelor's or diploma	753	71.6	549	72.9	204	27.1	
	Postgraduate (Master’s or PhD)	95	9.0	73	76.8	22	23.2	
Region	Northern Region	218	20.7	157	72.0	61	28.0	0.917
	Southern Region	165	15.7	125	75.8	40	24.2	
	Eastern Region	264	25.1	190	72.0	74	28.0	
	Western Region	169	16.1	123	72.8	46	27.2	
	Central Region	236	22.4	174	73.7	62	26.3	
Number of children	We are planning for	204	19.4	169	82.8	35	17.2	0.001
	1	111	10.6	80	72.1	31	27.9	
	2	117	11.1	92	78.6	25	21.4	
	3	143	13.6	103	72.0	40	28.0	
	4 or more	477	45.3	325	68.1	152	31.9	

Evaluation of Saudi Arabian parents' knowledge regarding developmental milestones' red flags

Table 2 reveals some interesting findings regarding parental knowledge of developmental milestones. There were higher levels of awareness in certain areas. For instance, a significant majority of parents (809, 76.9%) correctly identified that a child should be able to walk by 12 months. However, it is notable that only 309 (29.4%) parents correctly identified that a child should be able to crawl by nine months, while 290 (27.6%) recognized that a child should be able to sit without support by six months. Similarly, 293 (27.9%) parents correctly identified that a child should be able to sit with support by four months. These percentages indicate a lower level of knowledge in these specific areas of development. When it comes to pointing to a desired object, only 278 (26.4%) parents recognized that this milestone should be reached by nine months. On a positive note, 440 (41.8%) parents correctly identified six months as the age at which a child begins to respond to their own name.

**Table 2:** Evaluation of Saudi Arabian parents' knowledge regarding developmental milestones' red flags. *Correct response.

Questions		Count, *n*	%
Knowledge regarding gross motor milestones			
By which age should a child be able to crawl?	11 months	60	5.7
	9 months*	309	29.4
	7 months	636	60.5
	I don’t know	47	4.5
By which age should a child be able to sit without support?	4 months	16	1.5
	6 months*	290	27.6
	8 months	679	64.5
	I don’t know	67	6.4
By which age should a child be able to sit with support?	4 months*	293	27.9
	6 months	551	52.4
	8 months	154	14.6
	I don’t know	54	5.1
By which age should a child be able to pedal a tricycle?	3 years*	466	44.3
	4 years	270	25.7
	5 years	270	25.7
	I don’t know	46	4.4
By what age should a child be able to roll over in either direction?	6 months*	442	42.0
	4 months	425	40.4
	2 months	92	8.7
	I don’t know	93	8.8
By what age should a child be able to walk?	10 months	179	17.0
	12 months*	809	76.9
	8 months	32	3.0
	I don’t know	32	3.0
Knowledge regarding fine motor milestones			
By what age should a child be able to do a pincer grasp i.e. hold an object with index finger and thumb?	4 months*	417	39.6
	6 months	271	25.8
	8 months	287	27.3
	I don’t know	77	7.3
By what age should a child put objects in his/her mouth?	6 months*	792	75.3
	12 months	132	12.5
	36 months	56	5.3
	I don’t know	72	6.8
By what age should a child be able to transfer objects from one hand to the other?	12 months	334	31.7
	6 months*	228	21.7
	9 months	412	39.2
	I don’t know	78	7.4
Knowledge regarding social and help milestones			
By what age should a child be able to drink from a cup and use a spoon?	6 months	29	2.8
	1 year*	413	39.3
	2 years	569	54.1
	I don’t know	41	3.9
By what age should a child be able to smile spontaneously?	4 months	308	29.3
	6 months	175	16.6
	2 months*	532	50.6
	I don’t know	37	3.5
By what age does a child develop a fear of strangers?	4 months	229	21.8
	6 months*	332	31.6
	8 months	395	37.5
	I don’t know	96	9.1
By what age should a child be able to point to a desired object?	12 months	623	59.2
	6 months	55	5.2
	9 months*	278	26.4
	I don’t know	96	9.1
By what age should a child be toilet trained?	3 years*	436	41.4
	4 years	115	10.9
	2 years	469	44.6
	I don’t know	32	3.0
By what age does a child normally begin to recognize his/her caregiver?	12 months*	231	22.0
	18 months	134	12.7
	6 months	631	60.0
	I don’t know	56	5.3
Knowledge regarding language milestones			
By what age does a child begin to respond to his/her own name?	12 months	214	20.3
	6 months*	440	41.8
	9 months	349	33.2
	I don’t know	49	4.7
By what age does a child say his first word?	12 months	229	21.8
	6 months	381	36.2
	9 months*	392	37.3
	I don’t know	50	4.8
By what age should a child be able to vocalize (i.e., make audible responses/sounds) when talked to?	12 months	257	24.4
	6 months*	444	42.2
	9 months	288	27.4
	I don’t know	63	6.0
By what age should a child have a vocabulary of 50 or more words?	12 months	46	4.4
	18 months*	219	20.8
	24 months	686	65.2
	I don’t know	101	9.6
By what age should a child be able to give his/her full name and age?	3 years*	768	73.0
	1 year	27	2.6
	2 years	186	17.7
	I don’t know	71	6.7
By what age should a child be able to call his mother and father *mama* and *dada,* respectively?	12 months	537	51.0
	6 months	116	11.0
	9 months*	338	32.1
	I don’t know	61	5.8
By what age should a child be able to respond to simple instructions like “sit down” or “bring it here”?	12 months*	407	38.7%
	18 months	478	45.4%
	9 months	111	10.6%
	I don’t know	56	5.3%

The overall level of knowledge among study participants regarding developmental milestones' red flags

**Figure 1:**
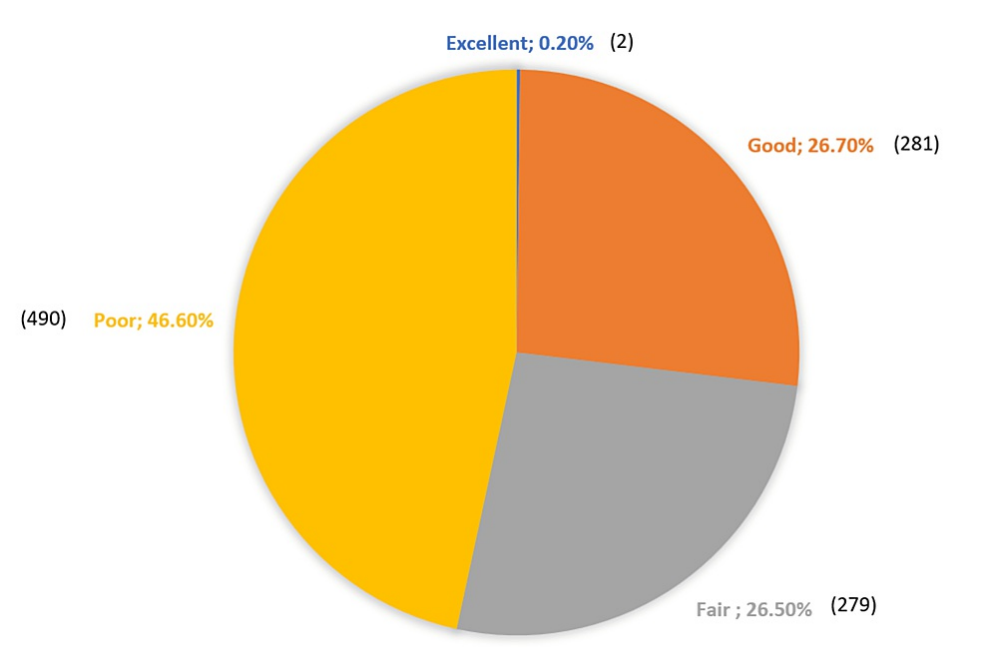
The overall level of knowledge among study participants regarding developmental milestones' red flags.

The results revealed that a small proportion (2, 0.2%) had excellent knowledge, while a larger percentage had good (281, 26.7%) or fair (279, 26.5%) knowledge. However, a significant portion (490, 46.6%) demonstrated poor knowledge in this area. The mean score of the participants was 8.6 ± 2.7 out of 22, indicating an average performance (Figure 1).

Evaluation of Saudi Arabian parents' attitudes regarding developmental milestones' red flags

Table 3 shows that approximately 542 (51.5%) parents actively sought information on developmental milestones themselves, indicating a significant interest in their child's development. However, 413 (39.3%) expressed agreement and 104 (9.9%) strongly agreed that pediatricians provided satisfactory and sufficient information in this regard, while 214 (20.3%) disagreed or strongly disagreed. Regarding developmental delay assessment based on family history, a proactive approach was observed, with 438 (41.6%) agreeing and 172 (16.3%) strongly agreeing. In terms of perceptions, 32.6% agreed or strongly agreed that delays in motor development indicated physical disability, while 28.3% agreed or strongly agreed that social and verbal development could lead to deafness or muteness. However, 38.3% disagreed or strongly disagreed with the latter statement.

**Table 3:** Evaluation of Saudi Arabian parents’ attitudes regarding developmental milestones' red flags.

Questions		Count, *n*	%
Have you ever looked up/sought information for children's developmental milestones yourself?	No	510	48.5
	Yes	542	51.5
Pediatricians (children's doctors) provide satisfactory and sufficient information regarding children's developmental milestones and their red flags.	Agree	413	39.3
	Strongly agree	104	9.9
	Disagree	123	11.7
	Strongly disagree	90	8.6
	Neutral	322	30.6
In case of a positive family history, will you get the developmental delay assessment done?	Agree	438	41.6
	Strongly agree	172	16.3
	Disagree	86	8.2
	Strongly disagree	72	6.8
	Neutral	284	27.0
You consider delays in motor development are a strong indication of physical disability.	Agree	277	26.3
	Strongly agree	66	6.3
	Disagree	297	28.2
	Strongly disagree	98	9.3
	Neutral	314	29.8
You consider that social and verbal development can lead to the child becoming deaf and/or mute .	Agree	236	22.4
	Strongly agree	62	5.9
	Disagree	275	26.1
	Strongly disagree	135	12.8
	Neutral	344	32.7

Evaluation of Saudi Arabian parents’ practices regarding developmental milestones' red flags

Table 4 reveals that a significant proportion of parents (641, 60.9%) reported visiting or consulting a pediatrician as per their child's needs, while only 176 (16.7%) visited a pediatrician more than twice a year. Regarding the time spent with their children, 503 (47.8%) parents reported spending more than eight hours daily. When it comes to identifying developmental delays, the majority of parents (64.6%) expressed a positive response toward undergoing a developmental delay assessment. Additionally, a considerable number of parents (77.7%) agreed or strongly agreed that they spend time interacting with their children to improve their language and social development. In terms of seeking assistance for developmental delays, a majority of parents (529, 50.3%) preferred consulting a developmental pediatrician.

**Table 4:** Evaluation of Saudi Arabian parents’ practices regarding developmental milestones' red flags.

Questions		Count, *n*	%
How many times do you visit/consult a pediatrician (children's doctor) in a year on average?	One to two times	137	13.0
	More than two times	176	16.7
	As per need	641	60.9
	None	98	9.3
How much time do you spend with your child daily?	1-2 hours	57	5.4
	3-4 hours	142	13.5
	5-8 hours	301	28.6
	<1 hours	49	4.7
	>8 hours	503	47.8
On identifying delays in any domain, will you get the developmental delay assessment done?	Agree	540	51.3
	Strongly agree	145	13.8
	Disagree	59	5.6
	Strongly disagree	50	4.8
	Neutral	258	24.5
Do you spend time interacting with the child with an intent to improve their language and social development?	Agree	490	46.6
	Strongly agree	327	31.1
	Disagree	34	3.2
	Strongly disagree	49	4.7
	Neutral	152	14.4
Who should one consult if their child has a developmental delay?	General pediatrician	256	24.3
	Pediatric neurologist	148	14.1
	Family physician	119	11.3
	Developmental pediatrician	529	50.3

## Discussion

Principal findings 

This cross-sectional survey sought to assess the knowledge, attitudes, and practices regarding childhood developmental milestones among 1,052 Saudi parents. The findings reveal serious knowledge deficiencies, particularly in understanding age-specific developmental trajectories, with notable gaps observed in domains such as gross motor skills. Despite interests and positive intentions toward active developmental monitoring, there are substantial parental awareness shortfalls that need to be addressed through public health initiatives promoting milestone competencies and fostering partnerships between physicians and caregivers [1].

Knowledge inadequacies

Significant knowledge gaps persisted in determining age-based expectations for multiple developmental achievements like crawling, walking, grasping objects, self-feeding, responding to names, pointing at desired items, and vocabulary production [1]. On average, only 29%-42% of Saudi parents accurately recognized timeline norms for such fundamental infancy and toddler-stage milestones. This aligned with preceding regional evidence indicating limited milestone cognition, especially concerning motor development [2]. Saudi studies have reported under 50% parental awareness regarding norms for skills like sitting, walking, and coordination. Correspondingly, the current analysis revealed only around 27%-30% correct responses for motor indicators like crawling, sitting unsupported, and crossing midline, demonstrating continued understanding deficiencies among parents. 

Interestingly, relatively higher knowledge was observed for social milestones like spontaneous smiling and stranger anxiety, with 50%-75% accuracy rates. Comparable socioemotional development insight has been previously documented among Saudi parents [4]. Nevertheless, considerable awareness gaps persisted even for associated child interaction-communication milestones such as responding to a name, vocalization, and word usage, with only 26-42% correctly identifying developmental schedules. Such enduring knowledge inadequacies emphasize the need for prompt awareness-enhancing interventions targeting improved parental understandings of early childhood growth patterns across domains. They also correspond to global evidence indicating a widespread lack of developmental timeline clarity among caregivers [5-7]. Studies conducted among parents across Europe, Asia, and United States have shown limited milestone knowledge, highlighting this as a ubiquitous public health concern warranting prioritization [8-10]. 

Overall, less than 1% of Saudi parents exhibited excellent, while over 46% showed poor comprehension of childhood developmental trajectories. Average scores amounted to only around 39% of correct responses regarding age-based expectations for language, motor, cognitive, and sociobehavioral competencies. This lack of parent clarity regarding what constitutes developmentally appropriate skills at different life stages carries significant detrimental implications. Parents' ability to recognize red flags and abnormal delays hinges on internalized reference intervals for when children should achieve functional developmental capacity in areas like mobility, speech, or socialization [11,12]. Misconceptions regarding age-appropriate competence can heighten caregiver anxiety and precipitate premature interventions [13-15]. But more critically, inaccurate outlooks can camouflage genuine delays and allow interventions to be initiated only after optimal neuroplasticity windows expire [14]. Hence, enhancing the accuracies of parental developmental schemata merits urgent policy accentuation. 

Sociodemographic variations 

Interestingly, milestone knowledge showed significant sociodemographic associations, with women and experienced parents demonstrating more excellent competencies [1]. Mothers generally tend to be more involved in directly nurturing and monitoring early childhood growth [16]. Previous evidence also indicates maternal education, employment, and planned conceptions predict higher developmental awareness. Correspondingly, current findings revealed nearly 90% of fathers showcasing unacceptable knowledge versus only 70% of mothers. Additionally, parents actively raising children exhibited higher consciousness than those merely planning, potentially reflecting experiential gains in deciphering maturation trajectories through parenting exposures. These insights highlight the need for better engaging fathers and first-time parents through health communication targeting enhanced milestone clarity. They also suggest knowledge levels to be dynamic over time, underscoring the value of longitudinal tracking to ascertain the enduringness of observed patterns.

Attitudes and practices 

Despite severe knowledge limitations, Saudi parents essentially showed positive orientations toward seeking information and intentionally fostering developmental gains in children [7]. Over 50% actively pursued milestones insights themselves and trusted pediatric guidance. Most also expressed willingness for formal screenings if risk factors existed, indicating constructive outlooks despite substantial consciousness constraints. Caregiver endorsement and optimistic intent for monitoring developmental well-being offer fertile foundations for effectively cultivating more excellent competencies through public education. 

Additionally, nearly half the parents spent over eight hours daily interacting with children to nurture developmental needs across domains. Such involved parenting has demonstrated the ability to buffer even high-risk children from deviations [6] substantially. Nevertheless, directing these nurturance efforts optimally requires comprehensive, precise outlooks on milestone expectations, which persisted as inadequate among surveyed Saudi caregivers [8]. Hence, enabling maximal returns from positive parenting through developmental timeline clarity enhancements remains imperative. 

Communication gaps

Although Saudi parents essentially placed confidence in pediatric roles for milestone guidance, only around half regarded current physician information as entirely satisfactory for establishing adequate developmental consciousness [9]. Previous evidence indicates that nearly 60% of parents show a willingness for more excellent information from healthcare providers [4]. Poor physician-parent dialogue around development monitoring and delays has been repeatedly implicated in late abnormality recognition [11]. Current findings corroborate the continuation of such communication gaps between caregivers and children's doctors that require urgent attention to achieve timely surveillance and intervention. 

Strengthening pediatric partnerships through clinical policies prioritizing detailed, repeated development data exchange could help bridge consciousness disconnects [12]. Standardized screening questionnaires for routinely tracking maturation across domains beyond infancy could enhance detection capacities [14]. Similarly, establishing ongoing milestone-focused counsel in community settings through local healthcare worker parenting support programs shows promise [13]. Furthermore, longitudinal tracking of caregiver knowledge and physician input patterns using health informatics can clarify specific discussion deficiencies that need particular accentuation. Overall, the revealed consciousness gaps and communication limitations signal the need for multi-pronged initiatives fostering pediatric-parent synergy toward securing optimal developmental potentials among Saudi children.

This cross-sectional study illuminates the persistently limited consciousness among Saudi parents regarding age-anchored expectations for childhood developmental trajectory across domains spanning motor, sociobehavioral, communication, and cognitive competence maturation. Over 46% of surveyed caregivers demonstrate poor overall knowledge, with less than 30% correctly recognizing timeline norms for fundamental milestones like mobility indicators, crawling, and sitting unsupported that provide foundations for learning and independence. Sociodemographic variations exist, with mothers and experienced parents showcasing greater accuracies compared to fathers and first-time caregiver groups, respectively. Nevertheless, serious consciousness inadequacies endure across subgroups.

These parental knowledge gaps can severely impact capacities to distinguish typical and atypical developmental patterns for prompt red flag recognition and intervention. However, positive outlooks prevail regarding high self-driven information search efforts, trust in pediatric support, and willingness for formal screenings if risks exist [15]. Most caregivers also dedicate considerable daily interaction time with children focused on nurturing multidomain growth needs. This constructive developmental monitoring intent offers fertile ground for effectively fostering greater milestone consciousness.

Recommendations

Parental understanding of age-specific developmental competencies and aligned expectations constitute the fundamental framework that shapes the ability to recognize red flags and make informed intervention decisions for children [1]. This study reveals critical knowledge deficiencies among Saudi parents regarding developmental milestones that demand urgent public health prioritization to secure child well-being.

Design targeted education programs for parents focused on enriching domain-specific developmental milestone consciousness, especially among high-risk groups like fathers and first-time caregivers. In addition, increase pediatric partnerships through healthcare policies mandating detailed developmental counseling and growth tracking records to achieve better physician-parent synergy.

Conduct periodic developmental milestone-centered communication campaigns via multimedia to sustain consciousness augmentation. Moreover, incorporate evidence-based, comprehensively mapped landmark expectations into nationwide parenting resource materials for universal accessibility and develop localized social support structures like community healthcare workers who can provide continued milestone-focused parenting guidance.

Limitations

While providing exciting baseline insights into Saudi parental developmental milestone knowledge and associated aspects, this study has certain limitations.

First, the cross-sectional correspondence-based design restricts causal analysis of relationships between identified knowledge, attitudes, practices, and actual parent developmental monitoring behaviors and intervention decisions over time. Longitudinal tracking can better elucidate predictive links. Secondly, convenience recruitment and reliance on self-reports pose risks of sample biases and social desirability influences, which may temper the generalizability of the results concerning the broader Saudi society. Randomized representative statewide samples and empirical practice validation through observations can augment reliability.

In addition, the sample excluded non-Saudi residents and parents unwilling to participate, forfeiting valuable supplementary perspectives. Incorporating diverse groups can aid cross-cultural comparisons and help address participation barriers. Healthcare provider parents were also exempt from recruitment, preventing appraisal of a pertinent subgroup's developmental knowledge strengths and limitations relative to nonmedical caregivers. Future exploration of this cohort can offer enriched analytical insights.

The developmental framework itself was limited, with only selective milestones and domains included given questionnaire constraints, rather than exhaustively mapping entire maturation trajectories. More comprehensive designs can enable holistic knowledge evaluation. The knowledge assessment methodology relied simply on multiple-choice questions rather than employing complex validated psychometric developmental cognition scales. Advanced instrument adoption can improve analytical accuracies. The reliance on self-reported practices without external validation risks inherent responder biases. Supplementing subject perspectives with empirical parenting behavior observations through ethnographic approaches can enhance reliability.

Associations between caregiver developmental knowledge and children's longitudinal growth patterns were not examined. Relating parental understanding to child outcomes can provide direct impact estimations to inform policies. Finally, underlying sociocultural determinants like parenting philosophies and family dynamics that can mediate developmental knowledge should have been explored, limiting contextual clarity. Qualitative probes can offer rich peripheral insights into milestone comprehension.

## Conclusions

This study offers a vital foundation for informing healthcare ecosystem initiatives aimed at cultivating precision in parental developmental milestone consciousness to secure Saudi children’s well-being. It adds further Saudi-centric evidence to the persistent worldwide public health challenge of caregiver knowledge gaps that fundamentally shape timely and effective abnormality response capacities. The outcomes emphasize the need for urgent prioritization of parental developmental consciousness augmenting through multilevel interventions targeting improved physician dialogue, public education campaigns, and high-risk subgroup focus. They also outline directions for enriched explorations of underlying and consequent factors that can help strategically mold developmental trajectory knowledge among parents for enabling impactful monitoring and intervention. Overall, the research signals imperative accentuation on consolidating parent developmental milestone competencies for optimizing early childhood growth.
